# Diffusion‐weighted whole‐body imaging with background suppression (DWIBS) is effective and economical for detection of metastasis or recurrence of lung cancer

**DOI:** 10.1111/1759-7714.13820

**Published:** 2021-01-21

**Authors:** Katsuo Usuda, Shun Iwai, Aika Yamagata, Yoshihito Iijima, Nozomu Motono, Munetaka Matoba, Mariko Doai, Sohsuke Yamada, Yoshimichi Ueda, Keiya Hirata, Hidetaka Uramoto

**Affiliations:** ^1^ Department of Thoracic Surgery Kanazawa Medical University Kahoku‐gun Japan; ^2^ Department of Radiology Kanazawa Medical University Kahoku‐gun Japan; ^3^ Department of Pathology and Laboratory Medicine Kanazawa Medical University Kahoku‐gun Japan; ^4^ Department of Pathophysiological and Experimental Pathology Kanazawa Medical University Kahoku‐gun Japan; ^5^ MRI Center Kanazawa Medical University Kahoku‐gun Japan

**Keywords:** DWIBS (diffusion‐weighted whole‐body imaging with background suppression), lung cancer, magnetic resonance imaging, metastasis, positron emission tomography‐computed tomography

## Abstract

**Background:**

Diffusion‐weighted whole‐body imaging with background suppression (DWIBS) is used for the diagnosis and staging of cancers. The medical cost of an MR examination including DWIBS is $123, which is 80% less expensive than the cost ($798) of F18‐fluorodeoxyglucose positron emission tomography/computed tomography (FDG‐PET/CT) examination.

**Methods:**

This study examined the efficacy of DWIBS for relapses after lung cancer resection. A total of 55 patients who had pulmonary resection of lung cancer, postoperative computed tomography (CT) every six months, and DWIBS and FDG‐PET/CT (every year) were enrolled in this study. If a metastatic lesion was detected on CT scan, DWIBS and FDG‐PET/CT were also used.

**Results:**

A total of 55 patients who underwent pulmonary resections for lung cancer, and had CT, DWIBS and FDG‐PET/CT examination during follow‐up after pulmonary resection were enrolled in this study. Lung cancer in 32 patients relapsed. Postoperative radiographic examinations revealed pulmonary metastases in 17 patients, bone metastases in seven, liver metastases in five, lymph node metastases in five, pleural metastases in four, metastases to the chest wall in two, brain metastases in two, adrenal gland metastasis in one, and renal metastasis in one. The mean apparent diffusion coefficient (ADC) value of the relapse was 0.9 to 1.70 × 10^−3^ mm^2^/s. The accuracy 0.98 (54/55) of DWIBS for detecting multiple metastatic lesions was likely to be higher than 0.94 (52/55) of CT or 0.94 (52/55) of FDG‐PET/CT, but there were no significant differences.

**Conclusions:**

DWIBS can detect multiple metastatic lesions throughout the entire body and differentiate malignancy from benignity in only one examination. DWIBS has benefits of diagnostic accuracy and is less expensive in medical costs for the detection of a relapse. DWIBS could potentially replace FDG‐PET/CT after lung cancer resection.

## Graphical abstract

DWIBS has benefits of diagnostic accuracy and is less expensive in medical costs for the detection of a relapse. DWIBS could potentially replace PET‐CT after lung cancer resection.

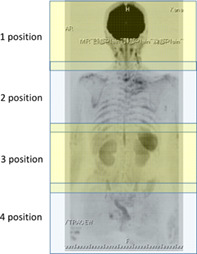

## INTRODUCTION

Although the diagnostic ability of magnetic resonance imaging (MRI) for evaluating lung cancer is considered equal to that of computed tomography (CT), the use of MRI for lung cancer was previously limited to invasion into the mediastinum or chest wall.^1^ However, recent methodological and technological advances in MRI, in particular diffusion‐weighted magnetic resonance imaging (DWI), prompted the Japanese lung cancer guidelines published in 2018 to suggest that MRI is an effective tool in the evaluation of benign and malignant nodules and masses.^2^

The technology of diffusion‐weighted whole‐body imaging with background suppression (DWIBS) which covers whole body MR examinations was described by Takahara *et al*. in 2004.^3^ The measured MR signal in DWI is attenuated by random molecular motion of (Brownian) water molecules. As for a tumor with high cell density, the extracellular space becomes narrow, and the movement of protons in the tumor become limited and its apparent diffusion coefficient (ADC) shows a lower number compared with that of normal tissue. Diffusion‐weighted imaging (DWI) can differentiate benign from malignant lesions in the lung,^4^, ^5^ thorax,^6^ prostate,^7^ breast,^8^ and liver.^9^ DWIBS means whole‐body DWI and is currently used for the diagnosis and staging of cancer, and for a response evaluation in the treatment for prostate cancer and breast cancer.^3^, ^10^ DWIBS can be applied not only to a few cancers (such as lymphoma and small cell tumors) that significantly restrict diffusion but also to many kinds of common cancers. Although DWI was reported to be better than CT for the response evaluation of chemo/radiotherapy in recurrent tumors of lung cancers,^11^ the benefit of DWIBS is still not widely known by respiratory physicians or by thoracic surgeons. To our knowledge, there has been only one article of diagnostic capability of DWIBS for lung cancer.^12^

The medical cost of examinations is an important issue. In Japan, it costs 10 000 yen ($88) for a CT scan, 18 000 yen ($157) for gallium scintigraphy, 22 000 yen ($192) for bone scintigraphy and 86 250 yen ($756) for a F18‐fluorodeoxyglucose positron emission tomography/computed tomography (FDG‐PET/CT).^13^ On the other hand, the medical cost of an MRI examination including DWIBS is 13 300 yen ($116) for MRI of 1.5T, or 16 000 yen ($140) for MRI of 3T, which is 80% less expensive than that of a FDG‐PET/CT. A DWI examination carries no risk of radiation exposure, does not need an exogenous contrast medium and patients do not have to fast before the examination.^14^ Furthermore an MRI examination with DWI requires less time (30 min) compared to FDG‐PET/CT (2 h).

The purpose of this study was to compare the diagnostic efficacy (sensitivity, specificity and accuracy) of DWIBS to that of CT and that of PET‐CT for metastasis, or recurrence after lung cancer resection, and to show the economic advantages of DWIBS.

## METHODS

### Eligibility

The Institutional Review Board in Kanazawa Medical University approved this study for examining patients with thoracic diseases with DWI (Approval No. I302). Due to contraindication, some patients with metal or pacemakers in their body or tattoos did not qualify for the study. After explaining the risks and benefits of the study informed consents were obtained from participating patients. All methods were carried out in accordance with the relevant guidelines and regulations of the Declaration of Helsinki.

### Patients

Over a period of 7 years from January 2012 to December 2018, the patients who underwent pulmonary removal for lung cancer, and agreed to participate in the trial were enrolled in this study. A total of 55 patients who underwent pulmonary resections for lung cancer, and had CT, DWIBS and FDG‐PET/CT examination during follow‐up after pulmonary resection were enrolled in this study (Table 1). For the follow‐up after pulmonary resection, CT examinations were performed in the chest area, or area of the chest‐pelvis every six months. DWIBS and FDG‐PET/CT examinations were performed annually. When patients had a recurrence at the six month CT interval, DWI and FDG‐PET/CT were also performed to gain further insight. There was no minimum size of lesions excluded from this study. All lesions that could be seen on CT scan were included in this study.

**TABLE 1:** Patient characteristics

Sex	Male	42
	Female	13
Age	55–88 (mean 70.6)	
Cell type	Adenocarcinoma	31
	Squamous cell carcinoma	15
	LCNEC	4
	AD‐SQ	3
	Carcinoid	1
	Large cell carcinoma	1
Pathological stage	IA	13
	IB	18
	IIA	5
	IIB	12
	IIIA	7
Procedure	Bilobectomy	1
	Lobectomy	44
	Segmentectomy	2
	Partial resection	8

Abbreviations: AD‐SQ, adenosquamous carcinoma; LCNEC, large cell neuroendocrine carcinoma.

There were 42 males and 13 females, with a mean age of 70.6 years (range: 55–88 years). There were 31 patients with adenocarcinomas, 15 with squamous cell carcinomas, four with large cell neuroendocrine carcinomas, three with adenosquamous carcinomas, one with carcinoid and one with large cell carcinoma.

### DWIBS (whole‐body DWI)

**FIGURE 1:**
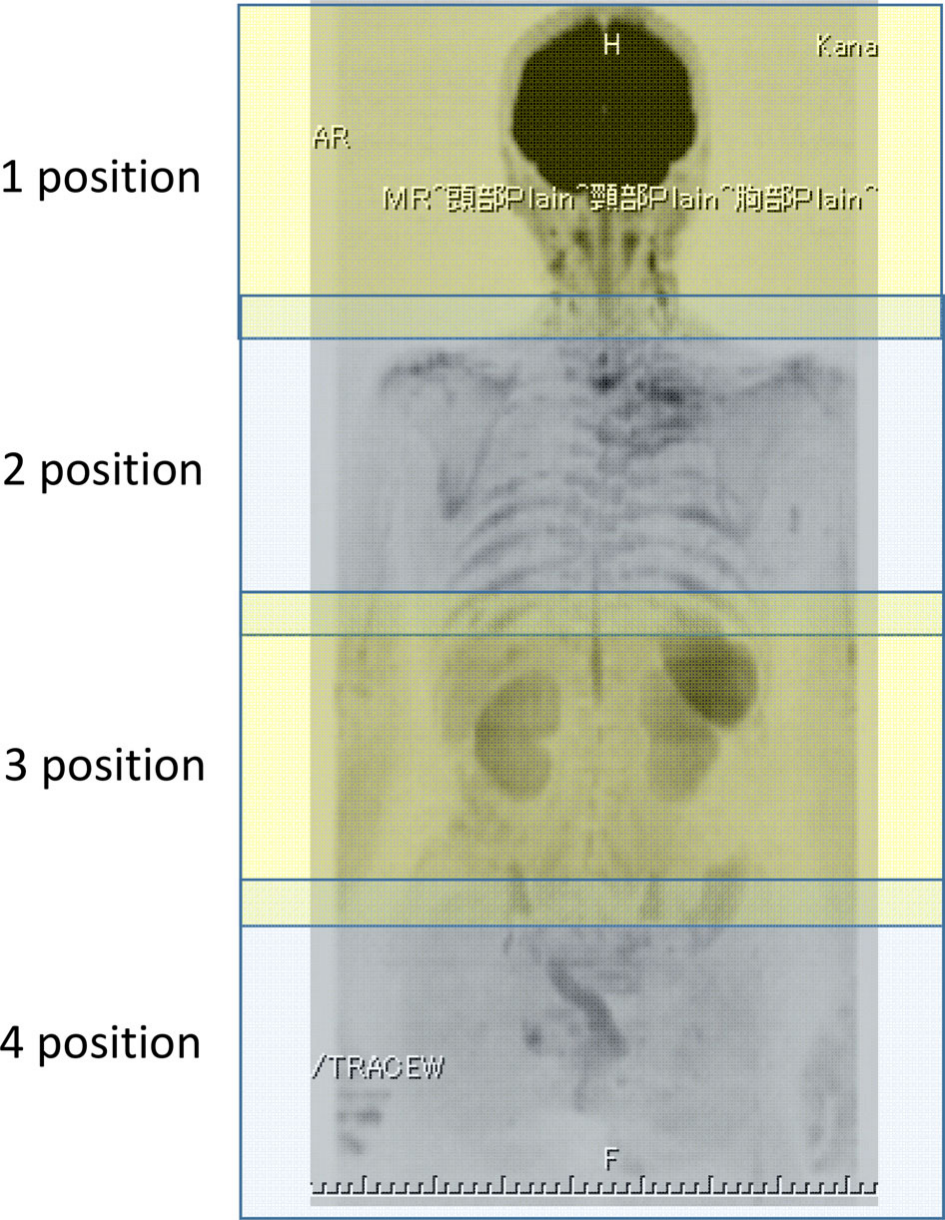
For diffusion‐weighted whole‐body imaging with background suppression (DWIBS), 4–5 positions were needed for each patient to scan the whole body. Each position required 4 min and 4–5 positions were repeated. T1WI of anatomical images is added for superposition of the images

A 1.5 T superconducting magnetic scanner (Magnetom Avanto; Siemens) was used for the MR image under single‐shot echo‐planar imaging (EPI) sequence with phased‐array coils including head, neck and body matrix coils. DWIBS were taken on STIR (short inversion‐time inversion recovery) method of fat suppression without breathing synchronization. When there were lesions in the thorax, chest DWI in the Prospective Acquisition CorrEction (PACE) method of respiratory triggering was added. DWIBS was performed in the axial plane with a slice thickness of 6‐mm with the following parameter: diffusion gradient encoding in three orthogonal directions; b value = 0 and 800 s/mm^2^; TR/TE/flip angle, 3000–4500/65/90; field of view, 350‐mm; matrix size, 128 × 128. For DWIBS, 4–5 positions were needed for each patient to scan the whole body. Each position required 4 min and 4–5 positions were repeated and T1WI of anatomical images added for superposition of the images (Figure 1). Parallel imaging of GRAPPA (generalized auto calibrating partially parallel acquisitions) in DWI/DWIBS was adopted. The receiver bandwidth of the EPI readout was 2480 (Hz/Pixel) in DWIBS. Each slice of DWI was taken seven times to ascertain the average. Total examination time was about 30 min. Afterwards coronal and sagittal planes were made by the rearrangement of the image of the axial plane. The lesion detection was done on the high b‐value images, and using that as a guide a two‐dimensional (2D) round or elliptical region of interest (ROI) was drawn on the ADC map by a licensed radiologist (M.D) with 27 years of MRI experience who was unaware of the patients' clinical data. Areas of necrosis were excluded from the ADC measurement. The procedure was performed three times and the minimum ADC value was determined. The radiologist (M.D.) and a pulmonologist (K.U.) analyzed the MRI data. A consensus was reached if there were any differences of opinion. The optimal cutoff value (OCV) of ADC for diagnosing malignancy in DWI was confirmed to be 1.70 × 10^−3^ mm^2^/s by the receiver operating characteristics curve as previously reported.^15^ Hilar, mediastinal lymph nodes or other metastatic lesions with an ADC value of the same or less than the OCV were defined as positive. Hilar, mediastinal lymph nodes or other metastatic lesions with an ADC value of more than the OCV or those that could not be detected on DWI were defined as negative.

### FDG‐PET/CT

A dedicated PET camera (SIEMENS Biograph Sensation 16) was used for FDG‐PET scanning before surgery. All patients fasted for 6 h before scanning. The dose of ^18^F‐FDG administered was 3.7 MBq/kg of bodyweight. After a 60 min uptake period, an emission scan was performed for 3 min per bed position and a whole‐body scan was obtained. Image reconstruction was done, and a two‐dimensional (2D) round region of interest (ROI) was drawn on a slice after detection of the highest count on the fused CT image by a radiologist (N.W.) with 36 years of PET‐CT experience who was unaware of the patients' clinical data. For the lesions with negative or faintly positive PET findings, the ROI was drawn on the fusion image with the corresponding CT. From those ROI, the maximum standardized uptake value (SUVmax) was obtained. The radiologist (N.W.) and one pulmonologist (K.U.) analyzed the FDG‐PET data. They eventually reached at the same consensus. The OCV of SUVmax for diagnosing malignancy in PET‐CT was confirmed to be 4.45 using the receiver operating characteristics curve as previously reported.^15^

### Statistical analysis

The computer software program StatView for Windows (Version 5.0; SAS Institute Inc. Cary, NC, USA) was used for statistical analysis. The data is expressed as the mean ± standard deviation. The sensitivity, specificity and accuracy of DWIBS, CT and FDG‐PET/CT were compared using McNemar's test. A *p*‐value of <0.05 was considered statistically significant.

## RESULTS

Of the 55 patients in the study, lung cancer in 32 patients relapsed, and pathological diagnoses of the relapses were determined in 16 patients. Radiological diagnoses of the relapses without pathological evidence were determined in the remaining 16 patients.

### Radiologic findings of metastatic lesions in DWIBS

#### Case 1. Large cell neuroendocrine carcinoma (pT2aN0M0)

**FIGURE 2:**
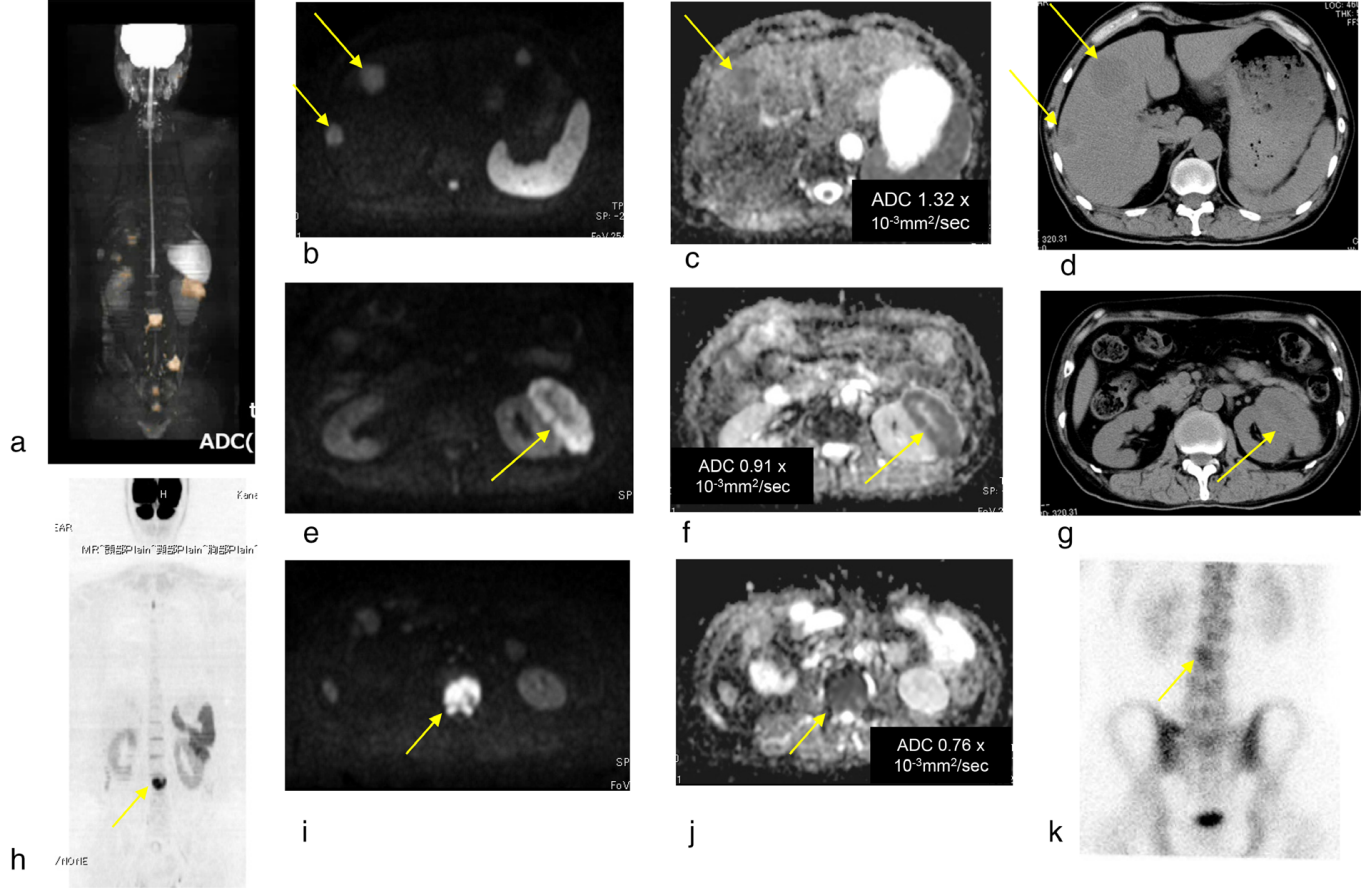
Case 1. Large cell neuroendocrine carcinoma (LCNEC) pT2aN0M0. (a) Whole body imaging of diffusion‐weighted whole‐body imaging with background suppression (DWIBS) show metastases in the liver, kidney, vertebra and ilium. (b) Multiple liver metastases were present on DWIBS, (c) apparent diffusion coefficient (ADC) map, and (d) CT. (c) The ADC of a liver metastasis was 1.32 × 10^−3^ mm^2^/s. Left renal metastasis was present on (e) DWIBS, (f) ADC map, and (g) CT. The ADC of the left renal metastasis was 0.91 × 10^−3^ mm^2^/s (f). (h, i) Metastases to lumbar vertebra is shown in DWIBS and (k) in bone scintigraphy. (j) The ADC of the one lumbar vertebra metastasis was 0.76 × 10^−3^ mm^2^/s. DWIBS showed the lumbar vertebra metastasis more clearly than bone scintigraphy

Whole body imaging of a DWIBS examination showed multiple metastases to liver, kidney, vertebra and ilium. It detected multiple metastases throughout the whole body. Metastatic lesions showed decreased diffusion of 1.7 × 10^−3^ mm^2^/s or less in ADC, compared to normal body tissue (Figure 2).

#### Case 2. Adenocarcinoma (pT2aN0M0)

**FIGURE 3:**
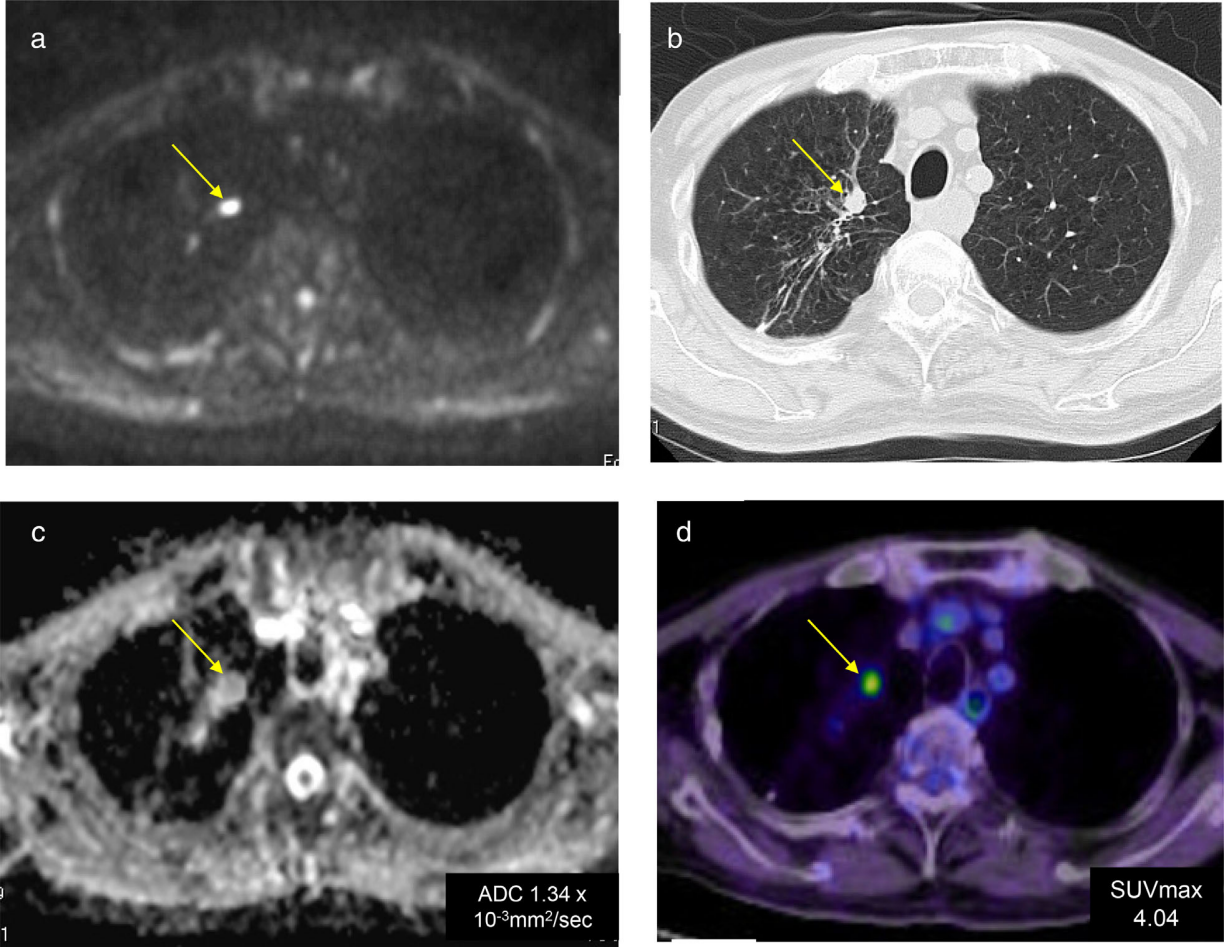
Case 2. Adenocarcinoma, pT2aN0M0. (a) Pulmonary metastasis was present on diffusion‐weighted whole‐body imaging with background suppression (DWIBS); (b) computed tomography (CT); and (d) FDG‐PET/CT. (c) The apparent diffusion coefficient (ADC) of pulmonary metastasis in ADC map was 1.34 × 10^−3^ mm^2^/s (d) The SUVmax of the pulmonary metastasis on FDG‐PET/CT was 4.04

DWIBS showed pulmonary metastasis as a lesion with decreased diffusion. The pulmonary lesion of right upper lobe was diagnosed as squamous cell carcinoma by transbronchial brushing cytology. The ADC of the lesion was documented to be 1.34 × 10^−3^ mm^2^/s. They were diagnosed as metastatic or recurrent lesions from the original lung cancer (Figure 3).

#### Case 3. Adenocarcinoma (pT2aN0M0)

**FIGURE 4:**
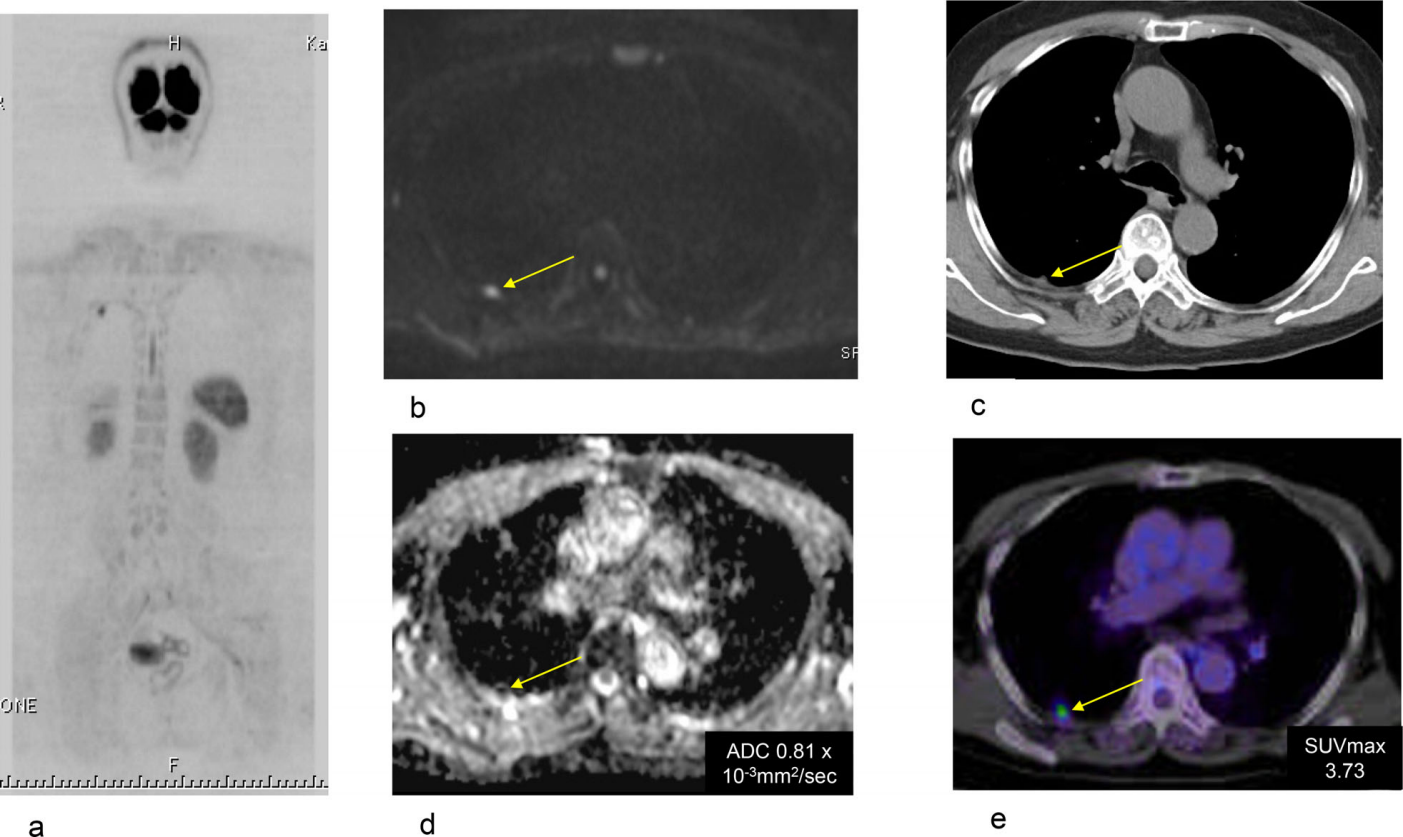
Case 3. Adenocarcinoma, pT2aN0M0. (a, b) Pleural metastasis was present in diffusion‐weighted whole‐body imaging with background suppression (DWIBS), (c) CT and (e) fluorodeoxyglucose positron emission tomography (FDG‐PET/CT). The apparent diffusion coefficient (ADC) of the pleural metastasis in the ADC map was 0.81 × 10^−3^ mm^2^/s (d). The SUVmax of the pleural metastasis in FDG‐PET/CT was 3.73 (e). A tiny metastatic lesion was detected on DWIBS

A pleural metastasis had a higher decreased diffusion on DWIBS. It was diagnosed as a metastasis from lung cancer by resection under thoracotomy (Figure 4).

#### Case 4. Squamous cell carcinoma (pT3N0M0)

**FIGURE 5:**
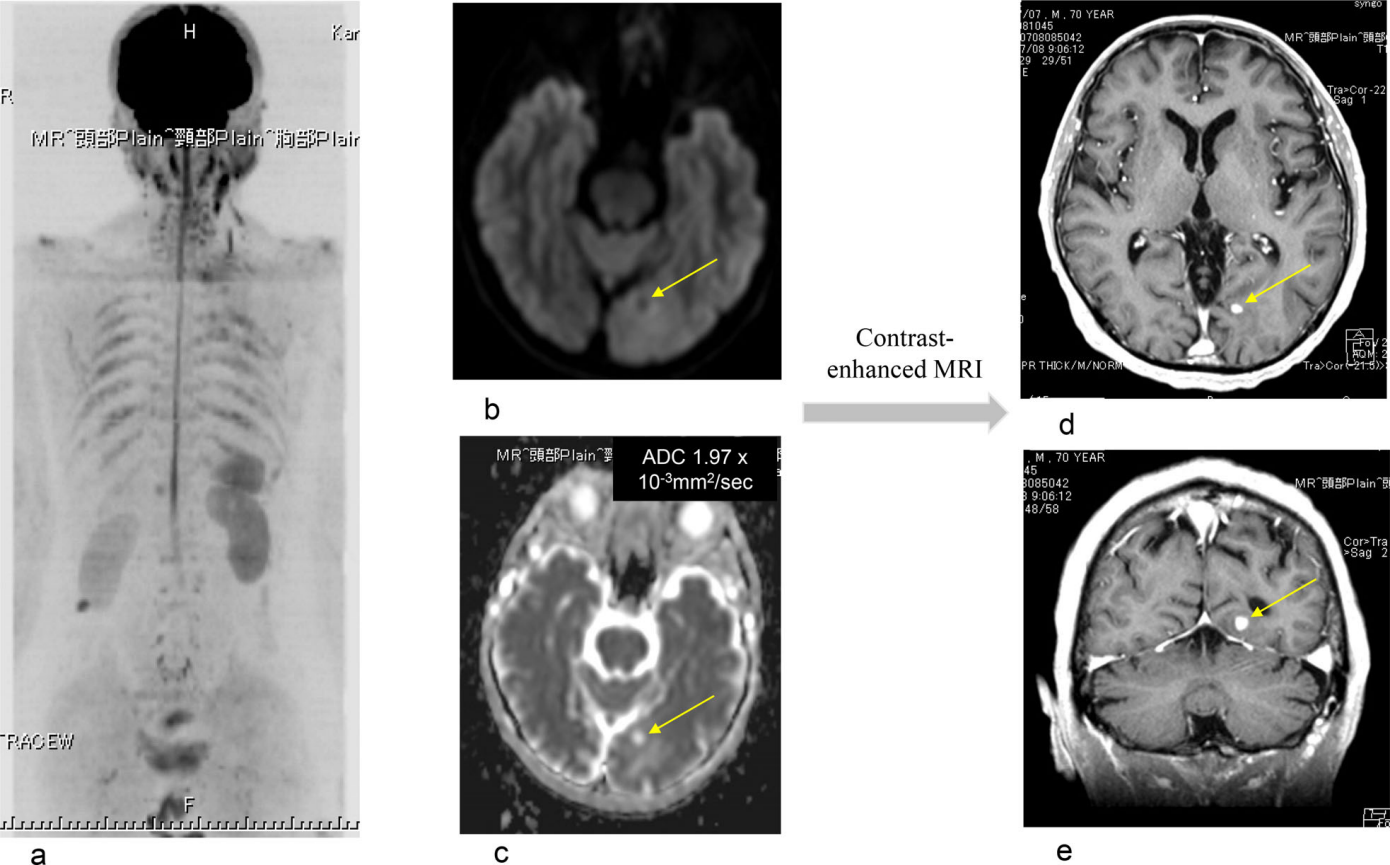
Case 4. Squamous cell carcinoma. pT3N0M0. (a) Whole body imaging in diffusion‐weighted whole‐body imaging with background suppression (DWIBS). (b) A tiny brain metastasis was presented in DWIBS. (c) The apparent diffusion coefficient (ADC) of the brain metastasis in the ADC map was 1.94 × 10^−3^ mm^2^/s, and was a false negative. (d, e) Contrast‐enhanced magnetic resonance imaging (MRI) determined a brain metastasis. The DWIBS‐enhanced lesion (ADC 2.04 × 10^−3^ mm^2^/s) at the level of the right kidney which was located in the abdominal wall and had not changed for one year was diagnosed as a benign neurogenic tumor

Serum CEA level was 16.5 ng/ml (normal <5.0 ng/ml) before surgery, decreased to 6.7 ng/ml after surgery, but later increased to 9.1 ng/ml. Recurrence was therefore strongly suspected. As there were no evidence of lesion recurrence on chest‐abdominal CT, DWIBS was performed in this patient and a tiny brain metastasis was found to be present. The ADC of the brain lesion in the ADC map was 1.94 × 10^−3^ mm^2^/s, and it was false negative. As there was no brain lesion on preoperative DWIBS, contrast‐enhanced brain MRI was performed which revealed the presence of an enhancing brain lesion. Finally it was confirmed to be a brain metastasis. The ADC of the original lung cancer of the brain metastasis differed by 0.885 to 2.01 × 10^−3^ mm^2^/s. The brain metastasis was speculated to come from a higher area of ADC of the primary lung cancer. The DWIBS‐enhanced lesion (ADC 2.04 × 10^−3^ mm^2^/s) at the level of the right kidney, which was located in the abdominal wall and had not changed for one year, was diagnosed as a benign neurogenic tumor (Figure 5).

DWIBS could be useful in the detection of brain metastasis. The cerebral lesion was not detected in CT or FDG‐PET/CT. DWIBS had an advantage for detecting cerebral lesions compared to CT or PET‐CT.

### Metastatic lesions and their ADCs

Examinations of CT, DWIBS and FDG‐PET/CT revealed pulmonary metastases in 17 patients, bone metastases in seven, liver metastases in five, pleural metastases in four, metastases to chest wall in two, brain metastases in two, lymph node metastases in five, adrenal gland metastasis in one, and renal metastasis in one (Table 2). ADCs (mean ± standard deviation × 10^−3^ mm^2^/s) of metastatic lesions were 1.29 ± 0.32 × 10^−3^ mm^2^/s in lung, 1.15 ± 0.47 × 10^−3^ mm^2^/s in bone, 1.14 ± 0.18 × 10^−3^ mm^2^/s in liver, 1.27 ± 0.34 × 10^−3^ mm^2^/s in pleura, 1.11 ± 0.19 × 10^−3^ mm^2^/s in chest wall, 1.64 ± 0.42 × 10^−3^ mm^2^/s in brain, 1.32 ± 0.33 × 10^−3^ mm^2^/s in lymph node, 1.04 × 10^−3^ mm^2^/s in adrenal gland, and 0.91× 10^−3^ mm^2^/s in kidney. On examination of DWIBS, all metastatic or recurrent lesions showed high signals on the image. The mean ADC of metastatic or recurrent lesions were 0.9 to 1.70 × 10^−3^ mm^2^/s. The majority of metastatic lesions except one brain metastasis were able to be judged as metastatic or recurrent lesions from lung cancer by DWIBS.

**TABLE 2:** Metastatic lesions and their ADCs

Recurrence or metastasis	No. of cases	ADC (×10^−3^ mm^2^/s)
Lung	17	1.29 ± 0.32
Bone	7	1.15 ± 0.47
Liver	5	1.14 ± 0.18
Pleura	4	1.27 ± 0.34
Chest wall	2	1.11 ± 0.19
Brain	2	1.64 ± 0.42
Lymph node	5	1.32 ± 0.33
Adrenal grand	1	1.04
Kidney	1	0.91

Abbreviation: ADC, apparent diffusion coefficient.

### Diagnostic efficacy of DWIBS, CT and FDG‐PET/CT

The sensitivities of detecting metastasis or recurrence were 0.97 (31/32) in DWIBS, 0.93 (30/32) in CT and 0.97 (31/32) in FDG‐PET/CT. The sensitivity of DWIBS was as high as that of FDG‐PET/CT. The sensitivity of DWIBS was likely to be elevated compared to that of CT, but not significant (*p* = 0.50). The specificity 1.0 (23/23) of DWIBS was likely to be higher than 0.95 (22/23) of CT or 0.91 (21/23) of FDG‐PET/CT, but not significant (DWIBS vs. CT [*p* = 1.0], DWIBS vs. FDG‐PET/CT [*p* = 0.47]). The accuracy 0.98 (54/55) of DWIBS was likely to be higher than 0.94 (52/55) of CT or 0.94 (52/55) of FDG‐PET/CT, but not significant (DWIBS vs. CT [*p* = 0.25], DWIBS vs. FDG‐PET/CT [*p* = 0.25]). There were no false positive cases using DWIBS (Table 3).

**TABLE 3:** Diagnostic efficacy based on type of examination

	CT	DWIBS	FDG‐PET/CT
False negative	2	1	1
False positive	1	0	2
True positive	30	31	31
True negative	22	23	21
Sensitivity	0.93 (30/32)	0.97 (31/32)	0.97 (31/32)
Specificity	0.95 (22/23)	1.0 (23/23)	0.91 (21/23)
Accuracy	0.94 (52/55))	0.98 (54/55)	0.94 (52/55)
Total cases	55	55	55

Abbreviations: CT, computed tomography; DWIBS, diffusion‐weighted whole‐body imaging with background suppression; FDG‐PET/CT, fluorodeoxyglucose positron emission tomography/computed tomography.

## DISCUSSION

### Diagnostic efficacy of DWIBS

The diagnostic efficacy of DWIBS compared to FDG‐PET/CT has been reported by some studies to have equal efficacy.^16^, ^17^, ^18^ However, since there is no radiation exposure with DWIBS, it is ideal for treating children.^19^ In gastrointestinal cancer, diagnostic efficacy of DWIBS was reported to be equivalent to that of FDG‐PET/CT.^20^, ^21^ In our study, the accuracy of using DWIBS (0.889) in the staging of N factor of lung cancer was probably higher than that (0.827) of FDG‐PET/CT + brain MRI, and that the accuracy (0.753) of the staging of lung cancer by DWIBS was also likely to be higher than that (0.691) of FDG‐PET/CT + brain MRI.^12^ Other studies have reported that diagnostic efficacy of DWIBS was better than that of FDG‐PET/CT.^14^, ^21^, ^22^ In ovarian cancer patients DWIBS has been reported to have higher diagnostic accuracy for primary tumors, peritoneal and distant staging compared to CT and FDG‐PET/CT.^23^ DWIBS showed better efficacy than FDG‐PET/CT for detecting hepatic and brain metastases because they were obscured by naturally high physiological FDG uptake of these organs.^14^, ^21^ On the other hand, for lymphoma assessment, diagnostic efficacy of DWIBS was equivalent to that of FDG‐PET/CT,^24^, ^25^ or inferior to that of FDG‐PET/CT.^26^ At present DWIBS seems to be an appropriate method for diagnosing malignant lymphomas.^27^ Assessment by DWI for patients with multiple hilar and mediastinal lymph nodes with FDG accumulation has been reported to be useful for distinguishing benignity and malignancy.^28^

DWI has been reported to be more accurate than FDG‐PET/CT in the detection of primary lesions and nodal assessment of non‐small cell lung cancers (NSCLCs).^29^ DWI has better diagnostic ability than PET in assessing pulmonary nodules and masses.^30^ DWI with ADC value and signal intensity can be useful for differential diagnosis of mediastinal lymph nodes.^31^ The pre‐eminence of DWI can be explained not only by DWI having fewer false‐positive results,^32^ but also DWI having fewer false‐negative results for N staging of NSCLC compared with FDG‐PET/CT.^29^ DWI could differentiate pleural effusion from pleural effusion with pleural dissemination or malignant pleural mesothelioma.^33^ We found that the functional evaluation of DWI was more accurate than the morphological evaluation of CT for the response evaluation of chemotherapy and/or radiotherapy to recurrent tumors of lung cancer.^11^ DWIBS (whole body DWI) could potentially replace FDG‐PET/CT after lung cancer resection.

The slice thickness (6‐mm) is standard for the examination of DWIBS. In this study size of metastatic lesions were 6‐ to 70‐mm in DWIBS and smaller metastatic lesions less than 6‐mm could be identified on not only DWIBS but also CT or FDG‐PET/CT. With lesions of less than 6‐mm being so difficult to detect we did not concentrate on them in this retrospective study. There is no problem for the slice thickness (6‐mm) on DWIBS for clinical usage.

### How to differentiate the lesion with MRI with DWI

In general, abscesses show high intensity in T2WI, extremely high intensity in DWI and extremely low ADC values. Acute ischemic stroke produces cytotoxic edema which connects to high intensity in DWI and its ADC is low. On the other hand, in lung cancer with central necrosis, its central necrosis shows high intensity in T2WI, but low intensity in DWI and its ADC is high. When differential diagnosis is difficult in DWIBS, enhanced CT, enhanced MRI or FDG‐PET/CT have to be performed. If necessary, transcutaneous biopsy will also be performed. When it is difficult to differentiate true recurrence/metastasis from stroke or abscess, enhanced brain MRI will be performed and lesions of true recurrence/metastasis will be enhanced.

### Medical costs

Medical costs have been increasing exponentially and show no signs of decreasing. Some of the rise in costs are due to increasing life spans and also by the expanding medical cost of examinations and various kinds of expensive therapies. In particular, the cost of FDG‐PET/CT is $756 and is extremely expensive. In general, medical examinations using radioisotopes are usually expensive. Therefore, DWIBS is an alternative examination for FDG‐PET/CT and has economic advantages compared to FDG‐PET/CT. Medical costs in Japan for FDG‐PET/CT or MRI are usually lower than the US. The average cost of an FDG‐PET/CT is $5750 (from $1250 to $9225) in the US, and $1800 in France. On the other hand, the average cost of an MRI is $2611 (from $1200 to $4000) in the US, and $280 in France, which illustrates that DWIBS has an economic advantage over FDG‐PET/CT.

### Limitations of MRI

DWI has two limitations. First, usually claustrophobic patients cannot undergo MRI. When they have to undergo MRI examination, they may require sedatives in order to deal with the situation. Second, patients who had metal or cardiac pacemakers in their body or tattoos should be excluded because these are contraindicated in MRI examination. In this study metal clips in patients caused no accidents because surgical metal tips were made with titanium which did not react in DWIBS.

### Limitations of the research

The issue we had from the beginning was the lack of patients willing to participate in the study. While not having a large sample size, this report does show DWIBS has the ability to diagnose lesions throughout the body. The patient number was limited because some patients did not agree to be in the trial. Due to the small sample size, further research in a larger cohort which includes patients at early stages of recurrence are necessary.

In conclusion, while FDG‐PET/CT is widely used for a follow‐up examination after resection of lung cancer, repeated follow‐up examinations impose a significant ionizing radiation burden, as well as economic cost. It has been proven that DWIBS after resection of lung cancer can detect metastatic or recurrent lesions of the entire body in only one examination at a reasonable medical cost. The diagnostic efficacy of DWIBS for metastatic or recurrent lesions is excellent when lesions present with decreased diffusion of ADC under 1.70 × 10^−3^ mm^2^/s. We recommend that routine follow‐up examinations after surgery of lung cancer consist of chest CTs (every six months) and DWIBS (every year). a DWIBS examination detects a lesion with decreased diffusion but cannot determine it is malignant or benign due to indeterminate or questionable lesions, additional enhanced MRI or CT examinations have to be performed for diagnostic confirmation of the lesion.
